# Investigation on Asphalt–Aggregate Interfacial Adhesion Under Hygrothermal Conditions Using Molecular Simulation

**DOI:** 10.3390/ma19214442

**Published:** 2026-10-22

**Authors:** Yan Li, Xinhao Sui, Rui Li, Xiaogang Guo, Rongquan Li, Jilong Yang

**Affiliations:** 1 School of Civil Engineering and Architecture, Nanyang Normal University, Nanyang 473061, China; 2 Larson Transportation Institute, Pennsylvania State University, University Park, PA 16802, USA; 3 College of Energy and Transportation Engineering, Inner Mongolia Agricultural University, Hohhot 010018, China; 4 School of Highway Engineering, Chang’an University, Xi’an 710064, China

**Keywords:** asphalt mixture, asphalt–aggregate interface, adhesion performance, molecular dynamics simulation, hygrothermal conditions

## Abstract

As a typical multiphase composite material, the asphalt mixture possesses an inherently weak zone at the asphalt–aggregate interface. This weak zone is especially vulnerable under hygrothermal conditions, where moisture and temperature jointly accelerate interfacial deterioration. In this study, molecular models of the asphalt–aggregate interface were constructed under both dry and wet conditions. The effects of aggregate type, temperature, and moisture, as well as their coupling effects, on interfacial adhesion performance were systematically investigated. The results indicated that the adhesion of asphalt binder to the three representative aggregate components decreased in the order of CaO, Al_2_O_3_, and SiO_2_. Within the temperature range of 5 °C to 65 °C, the adhesion work exhibited a non-monotonic trend, initially increased and then decreased, with a peak at 25 °C. Water molecules competitively adsorbed onto the aggregate surface upon entering the interface, hindering effective contact between asphalt molecules and aggregates. CaO showed the highest sensitivity to moisture, posing a high risk of moisture damage. Aggregate composition dominated interfacial adhesion, followed by moisture and then temperature. Moisture undermined adhesion by disrupting the interface contact, whereas temperature exerted a modulating effect that was more pronounced for SiO_2_ and Al_2_O_3_ at elevated temperatures, while CaO remained highly moisture-sensitive regardless of temperature. The coupled moisture–temperature effect further exacerbated interfacial failure, with high-temperature and high-humidity conditions causing the most significant damage to interfacial stability. This study reveals the adhesion performance and underlying mechanisms at the asphalt–aggregate interface from a microscopic perspective, clarifies the influence patterns and contribution weights of various factors, and thus provides a theoretical basis for optimizing the interfacial adhesion performance of asphalt mixtures.

## 1. Introduction

Asphalt mixtures are among the most widely used pavement materials in highway engineering owing to their excellent pavement performance, good ride comfort, and ease of construction [1]. However, because of the heterogeneous multiphase composite nature, the asphalt–aggregate interface is an inherently weak zone within the material [2]. The mechanical behavior and durability of this interface not only directly influence the load-bearing capacity of the asphalt mixture but also largely govern the macroscopic performance and long-term service life [3]. In the hot and humid regions of southern China, pavements are frequently exposed to high temperature and heavy rainfall during summer, subjecting the asphalt–aggregate interface to the combined effects of moisture ingress and high temperature [4,5]. Consequently, the interface is susceptible to adhesion failure, which readily triggers pavement distress, such as potholing, raveling, and cracking [6,7]. Given that the initiation and progression of these types of distress are intimately associated with interfacial behavior [8], a systematic investigation of asphalt–aggregate interfacial properties and the underlying interaction mechanisms is warranted. Such an investigation can provide a robust theoretical foundation for mitigating moisture-induced damage, delaying premature pavement deterioration, and enhancing pavement durability.

Conventional investigations of asphalt–aggregate interfacial behavior predominantly rely on macroscale tests, including the boiling water test, the freeze–thaw splitting test, and contact-angle measurements. Li et al. [9] reported that after freeze–thaw cycling, the interfacial adhesion between asphalt binder and aggregate decreased by more than 80%, while the stripping degree exceeded 90%. Luo et al. [10] found that the pull-off force gradually decreased with increasing relative humidity, accompanied by a transition in the interfacial failure mode from cohesive cracking to mixed cracking. Lei et al. [11] found that long-term aging reduced the surface free energy of asphalt binder and weakened its adhesion to aggregates. Zhang et al. [12] investigated the adhesion between castor oil-based bio-asphalt and aggregates using the boiling water test and contact-angle measurements, and found that bio-asphalt exhibited stronger adhesion to waste steel slag than to limestone. Guo et al. [13] reported that salt concentration, immersion duration, and the number of freeze–thaw cycles significantly affected interfacial pull-off tensile strength. Collectively, these experimental findings demonstrate at the macroscopic scale that environmental factors have a significant influence on interfacial adhesion performance. Nevertheless, the performance indicators derived from such conventional methods are inadequate for elucidating the underlying physical and chemical mechanisms that govern variations in adhesion behavior.

Recently, molecular simulation has seen growing application in asphalt materials research, providing an effective approach to addressing the limitations of conventional macroscopic methods [14]. As a simulation technique based on interatomic potentials, molecular simulation can characterize the material response under dynamic conditions at the atomic and molecular scales. By constructing molecular models of the asphalt–aggregate interface, the trajectories and interactions of molecules within the interfacial region can be directly tracked and analyzed using intermolecular forces and microstructural evolution [15]. This approach helps bridge the gap between macroscopic performance and microscopic mechanisms, offering a powerful analytical tool for investigating the behavior of the asphalt–aggregate interface. Wang et al. [16] reported that water molecules underwent competitive adsorption within the interfacial region, progressively displacing the lighter components of asphalt binder and forming hydrogen bonds with hydroxyl groups on the aggregate surface, thereby reducing the interfacial binding energy between asphalt binder and aggregate. Fu [17] found that water molecules progressively migrated toward the asphalt–aggregate interface and accumulated near the aggregate surface, leading to the formation of an interfacial water film. Fan et al. [18] demonstrated that aggregates rich in nepheline, chlorite, pyroxene, and olivine exhibited superior overall resistance to moisture-induced damage. Yao et al. [19] found that the adhesion energy of the water–aggregate interface exceeded that of the asphalt–aggregate interface, indicating that water molecules more readily displaced asphalt molecules and adsorbed onto the aggregate surface. Previous studies have largely focused on individual factors affecting interfacial adhesion performance, while systematic investigations into the coupled effects of aggregate composition, temperature, and moisture, as well as quantitative assessments of their relative contributions to interfacial stability, remain limited.

In this study, the interfacial adhesion behavior between asphalt binder and various aggregates was simulated using molecular simulation to elucidate the molecular interactions at the interface under both dry and wet conditions. The interfacial adhesion and the coupled effects of aggregate composition, moisture, and temperature were systematically investigated by analyzing relative concentration profiles and adhesion work. Furthermore, the relative contributions of these factors to interfacial stability were quantified. The findings are expected to provide a theoretical basis for improving the resistance of asphalt mixtures to moisture-induced damage and enhancing their performance under hot and humid environmental conditions.

## 2. Modeling and Simulation of Asphalt–Aggregate Interfacial Adhesion

### 2.1. Modeling of Asphalt Binder

Asphalt binder is a complex mixture composed of hydrocarbons with varying molecular weights and their non-metallic derivatives [20]. It is typically classified into several characteristic fractions based on differences in solubility and polarity. Currently, the four-component SARA (saturates, aromatics, resins, and asphaltenes) model is the predominant classification method, which divides asphalt binder into four fractions: saturates, aromatics, resins, and asphaltenes [21,22]. Within this model, asphaltenes and resins serve as the polar dispersed phase, uniformly distributed in the continuous medium consisting of saturates and aromatics, collectively maintaining the stability of the asphalt colloidal system [23].

**Figure 1:**
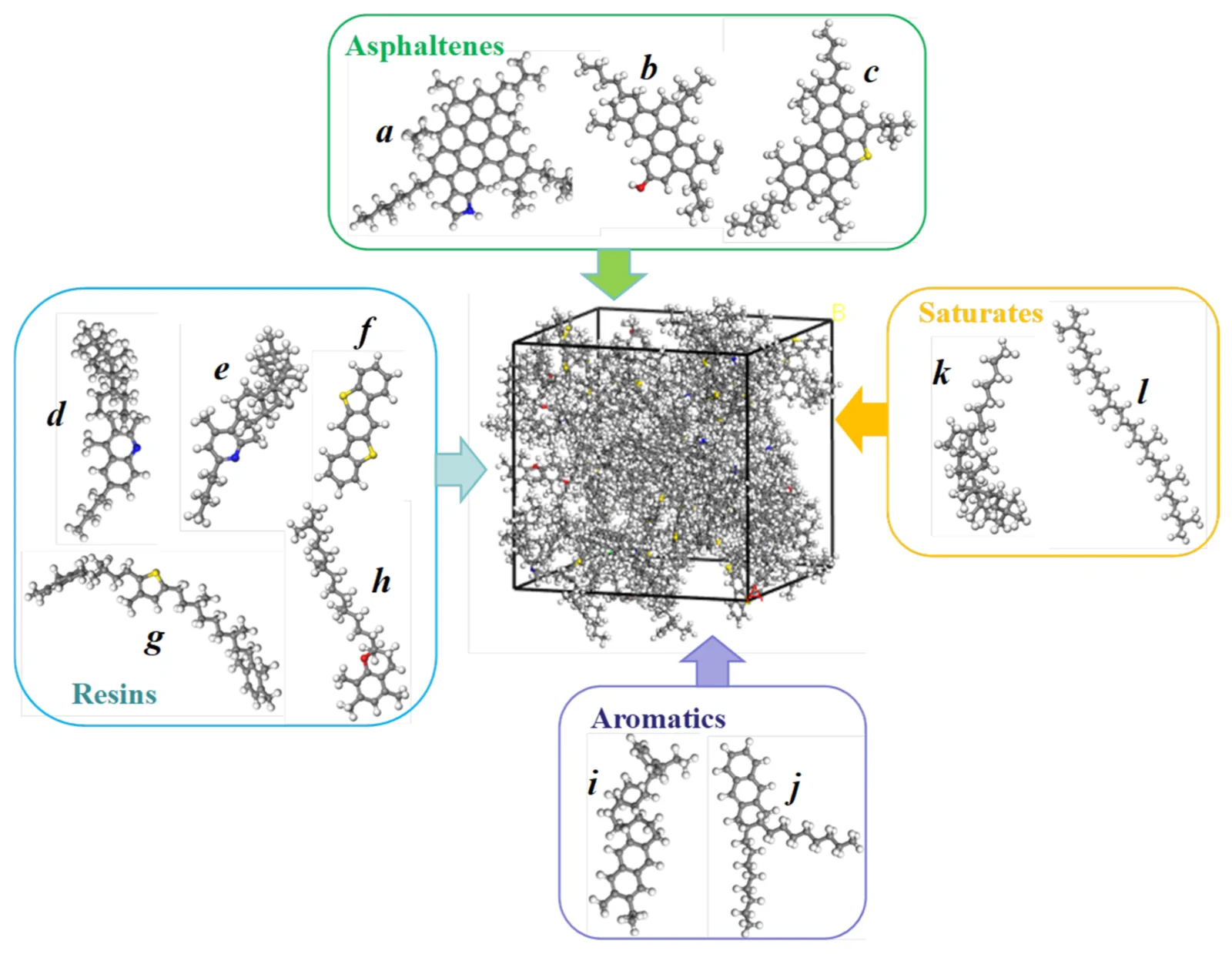
Representative molecules of SARA fractions and the corresponding molecular model of asphalt binder: (**a**–**c**) asphaltenes; (**d**–**h**) resins; (**i**,**j**) aromatics; (**k**,**l**) saturates.

The AAA-1 asphalt molecular model, proposed by the U.S. Strategic Highway Research Program (SHRP), is a representative model for characterizing the chemical composition of asphalt at the molecular scale based on the aforementioned SARA four-fraction classification framework [24]. This model comprises 12 representative molecules, whose molecular structures are shown in Figure 1, with detailed information, including chemical formulas and molecular numbers, listed in Table 1. The Amorphous Cell module in Materials Studio (version 2024) was employed to construct an amorphous unit cell by packing asphalt molecules into a cubic box with periodic boundary conditions. To relax the cell to an equilibrium configuration and eliminate initially unstable contacts, molecular dynamics simulations were conducted using the COMPASS II force field under the NPT ensemble for 100 ps, with a time step of 1 fs, a temperature of 25 °C, and a pressure of 1 atm. During the simulation, the volume of the asphalt binder model continuously decreased, and the density gradually increased, eventually stabilizing at approximately 1.03 g/cm^3^, which agrees well with the measured asphalt density (approximately 1.02–1.04 g/cm^3^), indicating that the model reached a stable equilibrium state and was suitable for subsequent simulations.

### 2.2. Modeling of Aggregates

**Figure 2:**
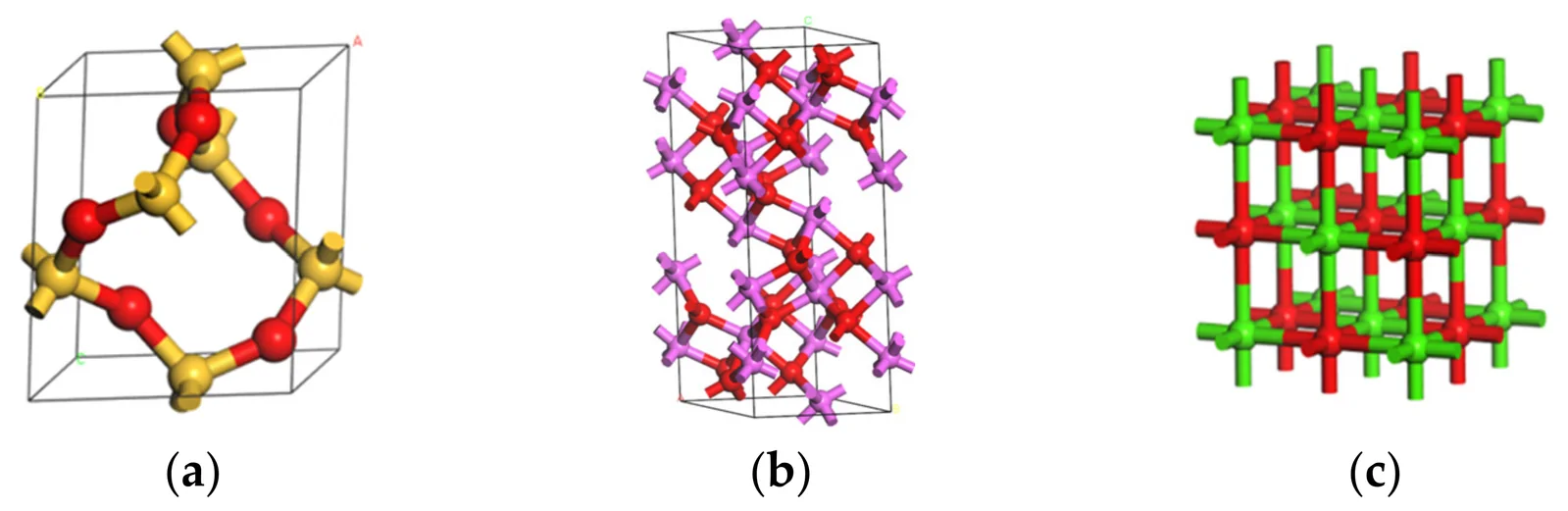
Unit cells of the representative oxides: (**a**) SiO_2_; (**b**) Al_2_O_3_; (**c**) CaO.

The physical and mechanical properties of aggregates are mainly determined by their mineral composition, with major chemical components including SiO_2_, Al_2_O_3_, CaO, Fe_2_O_3_, and MgO [25,26,27]. To investigate the effects of representative oxide surfaces on asphalt–aggregate interfacial behavior, SiO_2_, Al_2_O_3_, and CaO were selected and modeled separately under consistent simulation conditions, enabling a controlled comparison of their interactions with asphalt binder. Their unit cell structures are shown in Figure 2.

**Figure 3:**
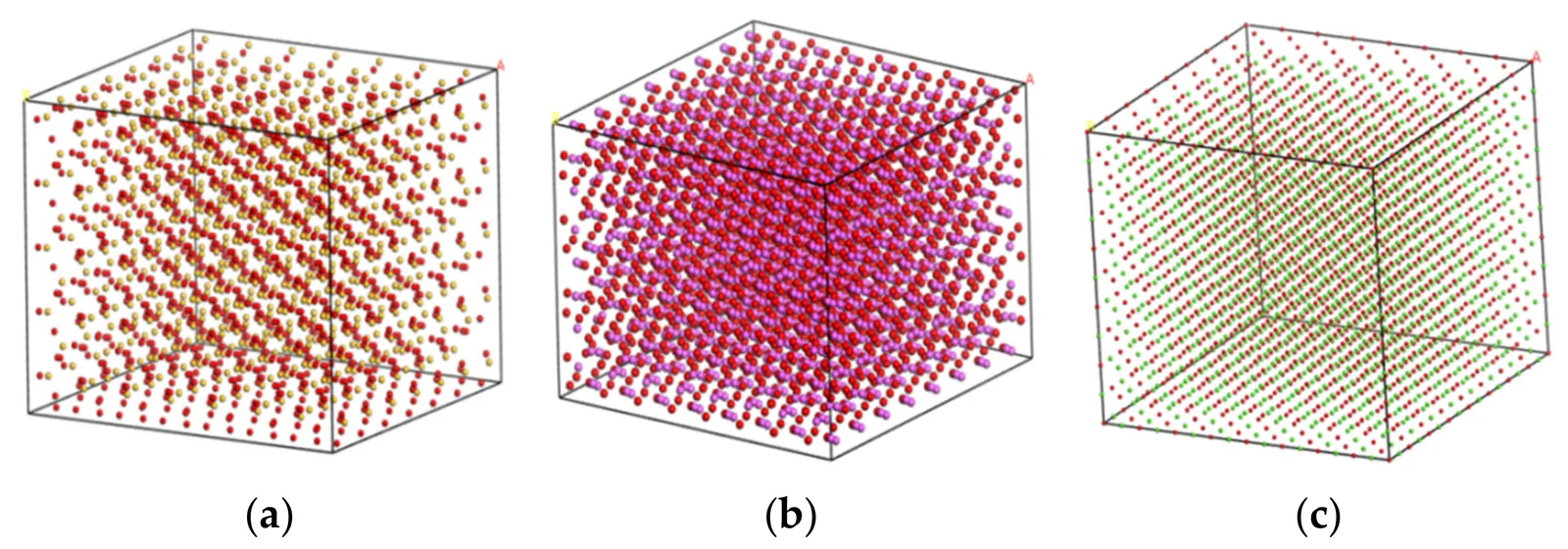
Molecular models of the representative oxides: (**a**) SiO_2_; (**b**) Al_2_O_3_; (**c**) CaO.

The initial unit cells of SiO_2_, Al_2_O_3_, and CaO were cleaved along the (0 1 0), (0 0 1), and (0 0 1) planes, respectively, yielding a Si-terminated SiO_2_ surface, an Al-terminated Al_2_O_3_ surface, and an O-terminated CaO surface. A vacuum layer was then introduced along the z-direction to separate periodic images and construct slab models with free surfaces. To obtain sufficiently large interfacial regions and reduce finite-size effects arising from periodic boundary conditions, the resulting slabs were further expanded into supercells. No surface hydroxyl groups were introduced into the initial models, and neither water dissociation nor surface hydroxylation was explicitly modeled. In order to eliminate residual internal stress, geometry optimization was performed with the bottom layers fixed and the surface layers allowed to relax. The final molecular models of the representative oxides are shown in Figure 3.

### 2.3. Modeling of Water

**Figure 4:**
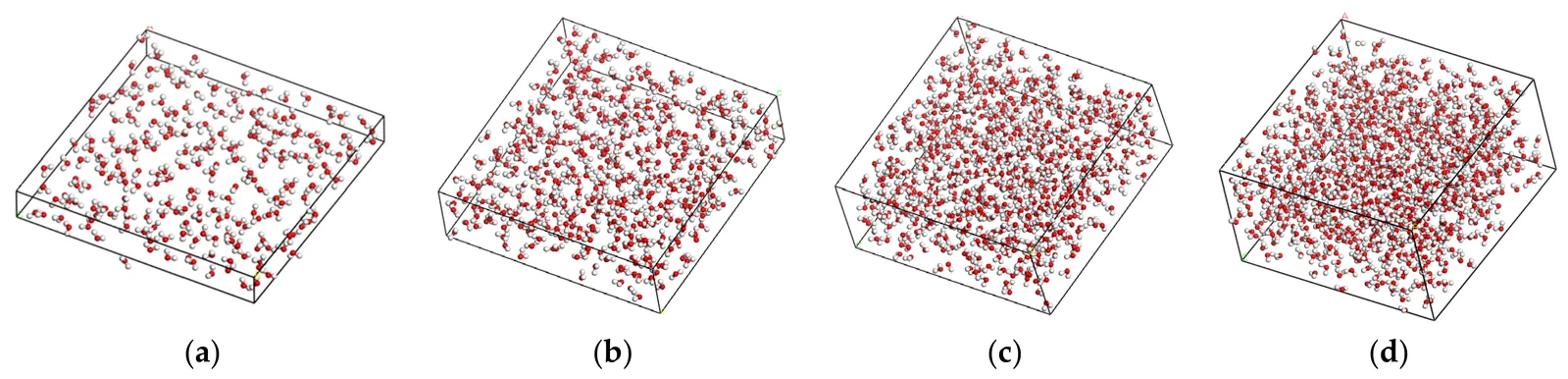
Molecular models of water with different thicknesses: (**a**) 5 Å; (**b**) 10 Å; (**c**) 15 Å; (**d**) 20 Å.

Water molecular models were constructed using the Amorphous Cell module and then optimized through energy minimization, followed by relaxation in the NPT ensemble to better approximate the state of real water under ambient conditions. Using this procedure, four water models were obtained by varying the number of water molecules, corresponding to water films with thicknesses of 5 Å, 10 Å, 15 Å, and 20 Å, as shown in Figure 4.

### 2.4. Modeling of Asphalt–Aggregate Interface

**Figure 5:**
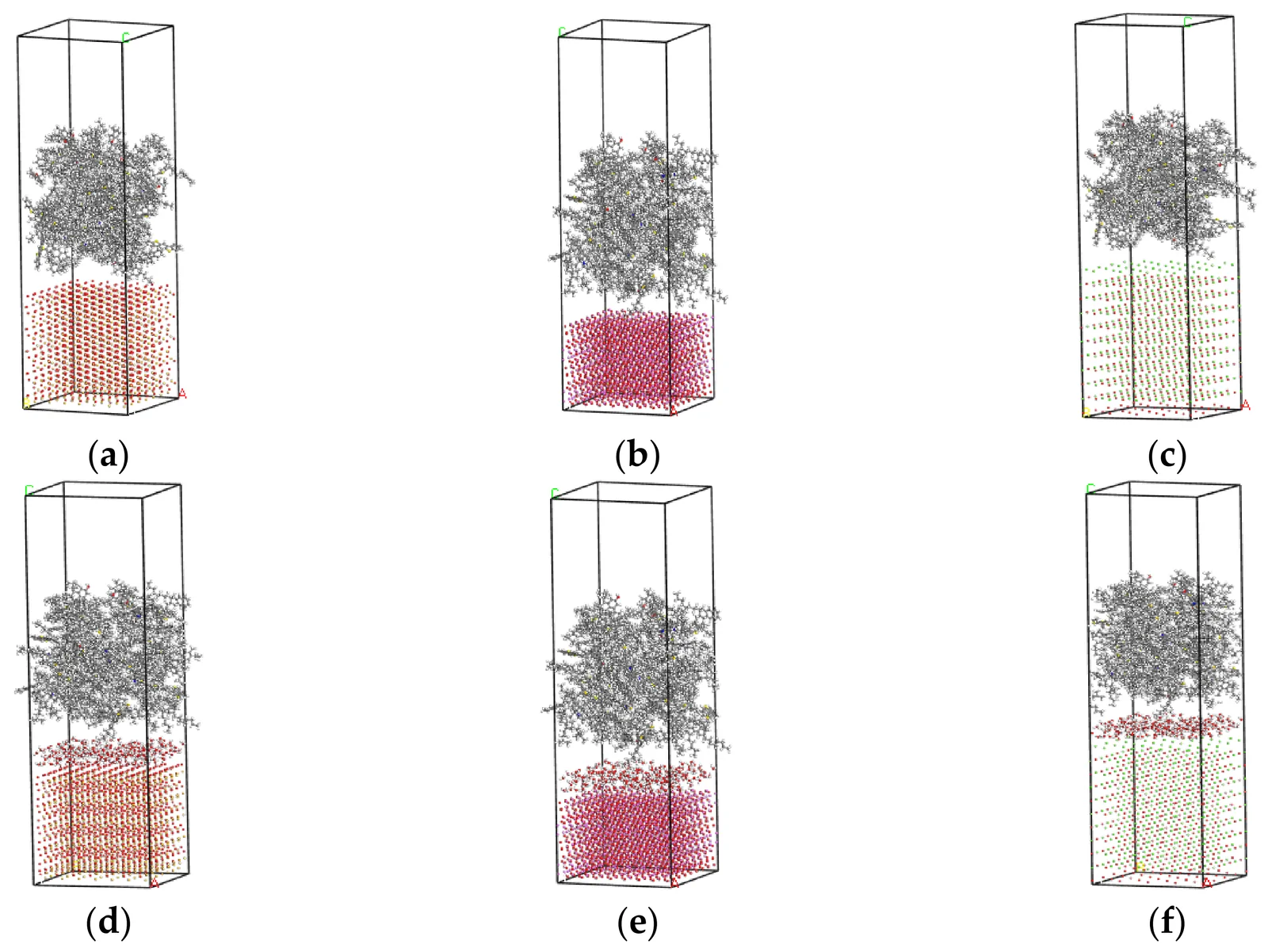
Molecular models of asphalt–aggregate interface under dry and wet conditions: (**a**) asphalt–SiO_2_; (**b**) asphalt–Al_2_O_3_; (**c**) asphalt–CaO; (**d**) asphalt–water–SiO_2_; (**e**) asphalt–water–Al_2_O_3_; (**f**) asphalt–water–CaO.

The asphalt–aggregate interface models were established using the Build Layer module considering both dry and wet conditions. The dry interface model consisted of the aggregate layer and the asphalt layer, as shown in Figure 5a–c. In the wet interface model, a water film was placed between the asphalt layer and the aggregate layer to represent the presence of interfacial moisture, as depicted in Figure 5d–f. Interface models containing water films with different thicknesses (5 Å, 10 Å, 15 Å, and 20 Å) were also established. Water permeation through the asphalt binder was not modeled because it exceeded the accessible simulation time scales, and this study focused on the effect of water film thickness rather than permeation.

Aggregates remain in a solid state within the pavement service temperature range, and their internal atomic thermal motion is extremely weak, allowing them to be approximated as fixed rigid structures. Accordingly, positional constraints were imposed on the aggregate-layer atoms during the simulation to accurately reflect their rigid characteristics and reduce computational cost.

Molecular simulations were performed on the asphalt–aggregate interface models in the NVT ensemble, with a total simulation time of 100 ps and an integration time step of 1.0 fs. To cover the pavement service temperature range, simulations were conducted at 5 °C, 25 °C, 45 °C, and 65 °C, with temperature controlled by a Nosé–Hoover thermostat. Five independent runs were performed for each interface model at the four temperatures. The reported interfacial properties were averaged over the independent runs.

### 2.5. Evaluation Methods for Interfacial Adhesion Work

The adhesion work is a key parameter for evaluating the interfacial adhesion between asphalt binder and aggregate. It is defined as the work required to separate a unit area of the interface, and its calculation method is given in Equation (1). A positive value of adhesion work indicates that energy input is required to separate the interface, and a larger positive value corresponds to a greater energy requirement for separation, thus indicating better interfacial adhesion performance. A negative value, on the other hand, indicates that the interface is in an unstable state and is prone to spontaneous separation.

(1) $$W_{ad}=\frac{{E_{agg}+E_{asp}-E}_{total}}{A}$$

where *W_ad_* is the adhesion work of the asphalt–aggregate interface, *E_agg_* is the energy of the aggregate layer, *E_asp_* is the energy of the asphalt layer, *E_total_* is the total energy of the interface model, and *A* represents the interfacial contact area.

The wet interface model consisted of three phases: asphalt binder, water, and aggregate. By treating the water layer and the aggregate layer as an integrated composite layer, the adhesion work between the asphalt binder and this composite layer can be calculated and defined as the wet-state adhesion work, as given in Equation (2).

(2) $${W^{\prime }}_{ad}=\frac{{{E^{\prime }}_{agg+wat}+{E^{\prime }}_{asp}-E^{\prime }}_{total}}{A}$$

where *W′_ad_* is the adhesion work of the asphalt–aggregate interface under wet conditions, *E′_agg+wat_* is the energy of the combined aggregate and water layers under wet conditions, *E′_asp_* is the energy of the asphalt layer under wet conditions, *E′_total_* is the total energy of the interface model under wet conditions, and *A* represents the interfacial contact area.

## 3. Results and Discussion

### 3.1. Configurational Evolution Analysis

**Figure 6:**
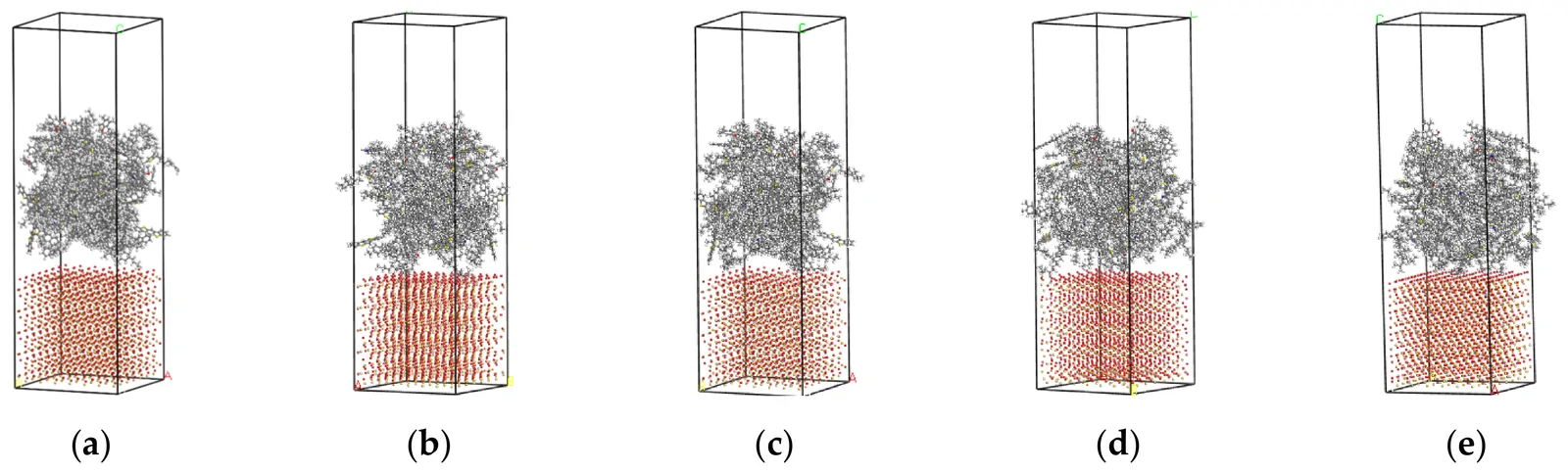
Configuration evolution of asphalt–SiO_2_ interface: (**a**) 0 ps; (**b**) 25 ps; (**c**) 50 ps; (**d**) 75 ps; (**e**) 100 ps.

**Figure 7:**
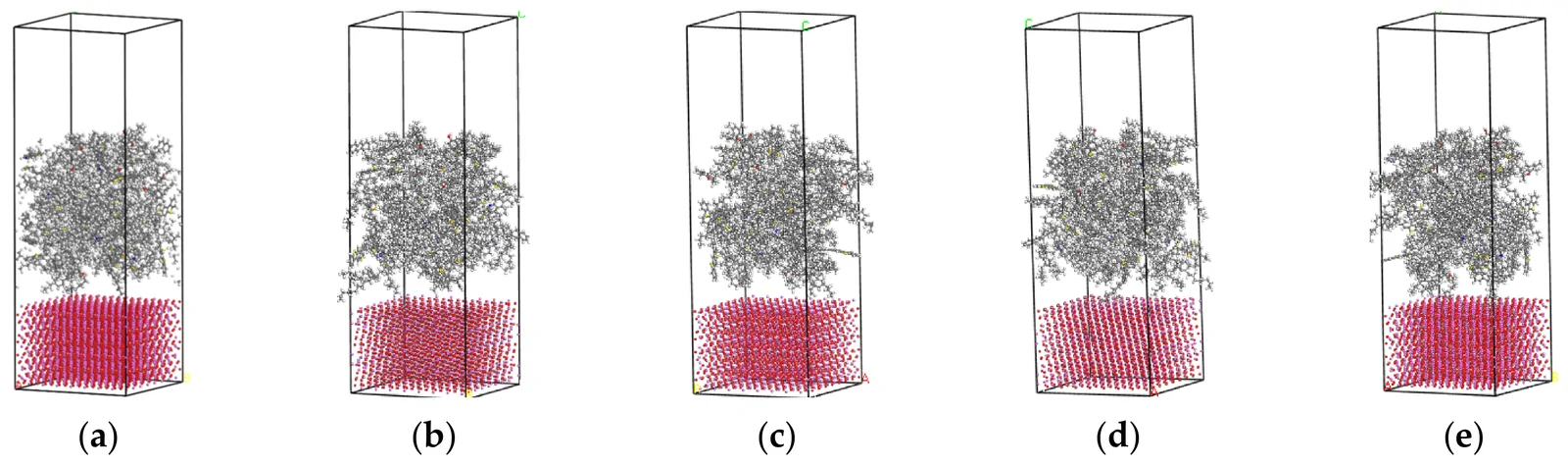
Configuration evolution of asphalt–Al_2_O_3_ interface: (**a**) 0 ps; (**b**) 25 ps; (**c**) 50 ps; (**d**) 75 ps; (**e**) 100 ps.

**Figure 8:**
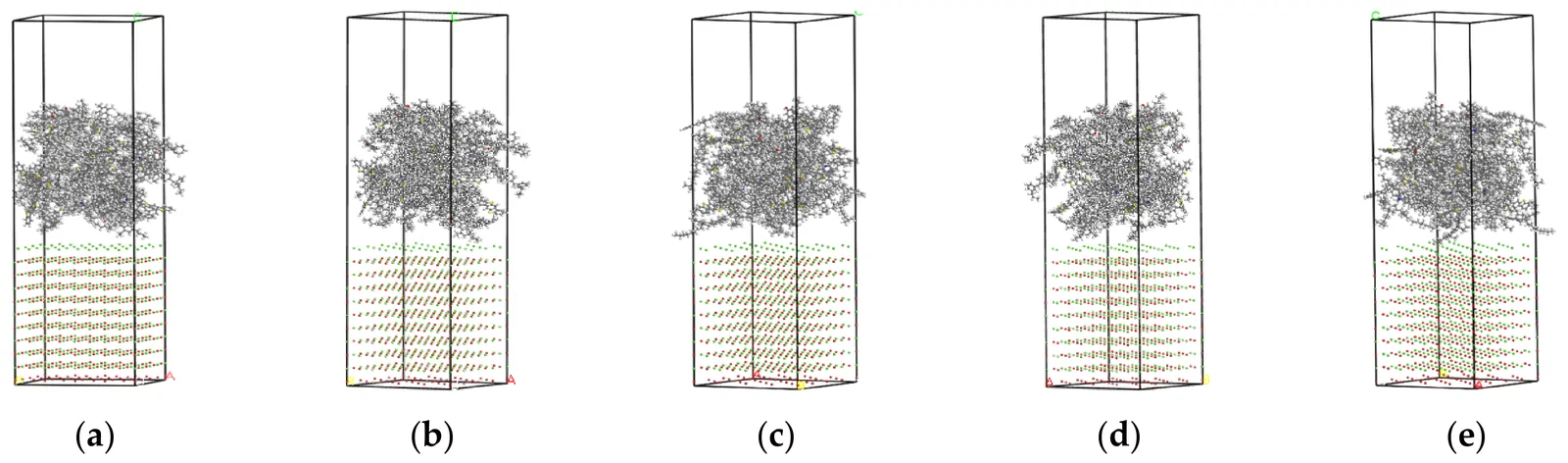
Configuration evolution of asphalt–CaO interface: (**a**) 0 ps; (**b**) 25 ps; (**c**) 50 ps; (**d**) 75 ps; (**e**) 100 ps.

Figure 6, Figure 7 and Figure 8 present the instantaneous configurations of the asphalt–aggregate interface under dry conditions during the simulation process. At the initial stage (0–25 ps), asphalt molecules were dispersed in a disordered manner within the interfacial region, maintaining a relatively large distance from the aggregate surface. As the simulation proceeded (50–75 ps), the molecular chains gradually underwent conformational adjustment, migrating toward and extending along the aggregate surface. By 100 ps, most asphalt molecules had come sufficiently close to the aggregate surface, forming a uniform interfacial contact layer. This configurational evolution clearly demonstrated that the aggregate surface exerted a thermodynamically driven spontaneous adsorption effect on asphalt molecules.

**Figure 9:**
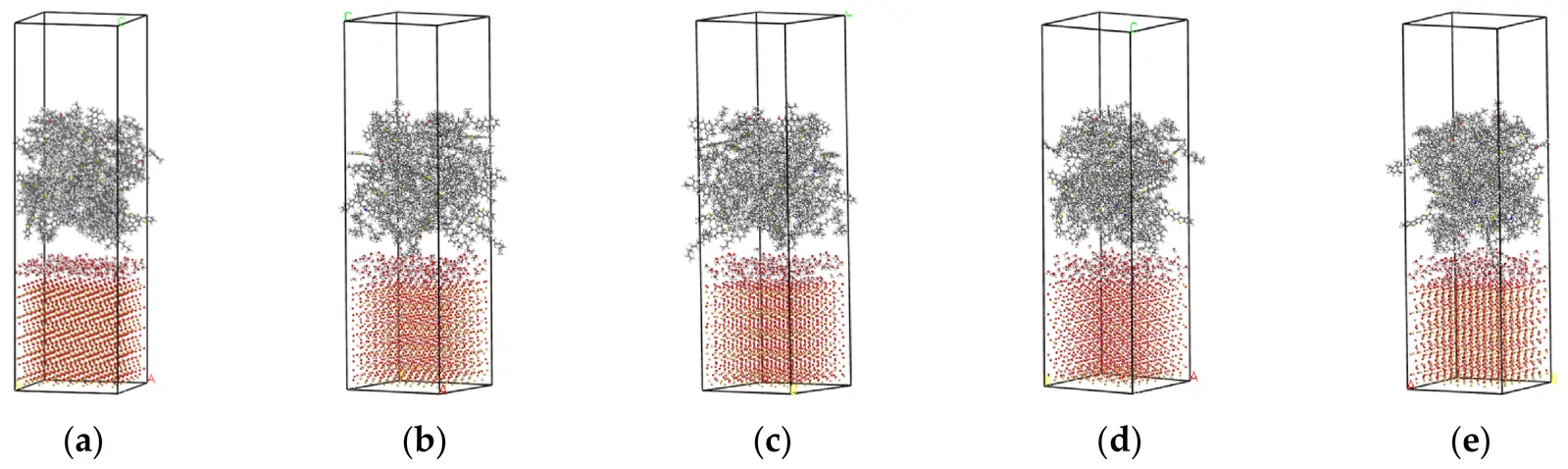
Configuration evolution of asphalt–water–SiO_2_ interface: (**a**) 0 ps; (**b**) 25 ps; (**c**) 50 ps; (**d**) 75 ps; (**e**) 100 ps.

The instantaneous configurations of the asphalt–aggregate interface under wet conditions at 0, 25, 50, 75, and 100 ps are shown in Figure 9. Throughout the simulation, water molecules remained at the interface, forming a continuous barrier layer. Consequently, instead of migrating toward the aggregate surface, asphalt molecules exhibited a clear tendency to move away from it, indicating that water significantly inhibited asphalt adsorption onto the aggregate.

**Figure 10:**
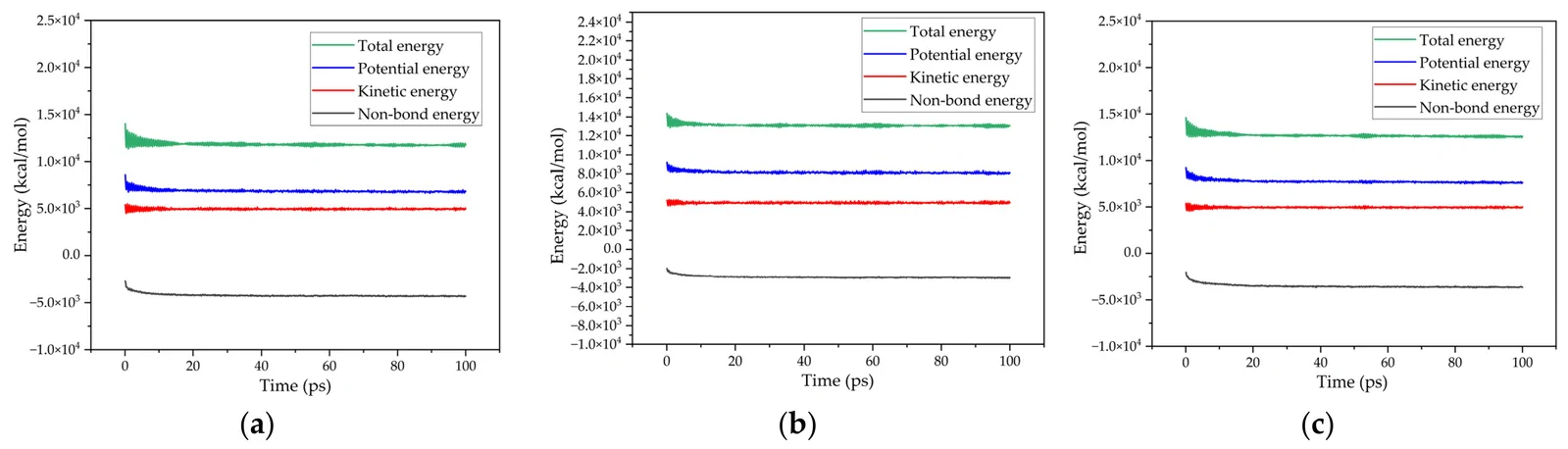

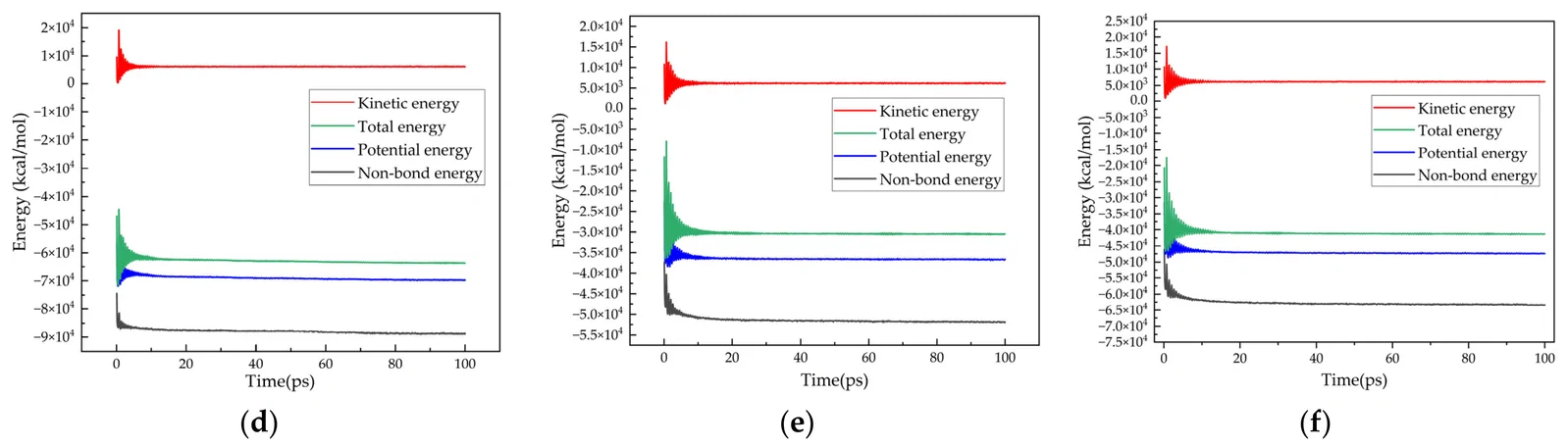
Energy evolution of interfacial adhesion simulation: (**a**) asphalt–Al_2_O_3_; (**b**) asphalt–SiO_2_; (**c**) asphalt–CaO; (**d**) asphalt–water–Al_2_O_3_; (**e**) asphalt–water–SiO_2_; (**f**) asphalt–water–CaO.

Figure 10 presents the energy evolution of the asphalt–aggregate interface during the simulations. All interfaces exhibited pronounced energy fluctuations during the initial stage, followed by progressively attenuated fluctuations. In the later stages of the simulations, the energy fluctuations remained small, and no systematic drift was observed, indicating that the systems had converged within the 100 ps simulation period. Therefore, the final configurations were suitable for subsequent adhesion performance analysis.

### 3.2. Relative Concentration Distribution Profiles

**Figure 11:**
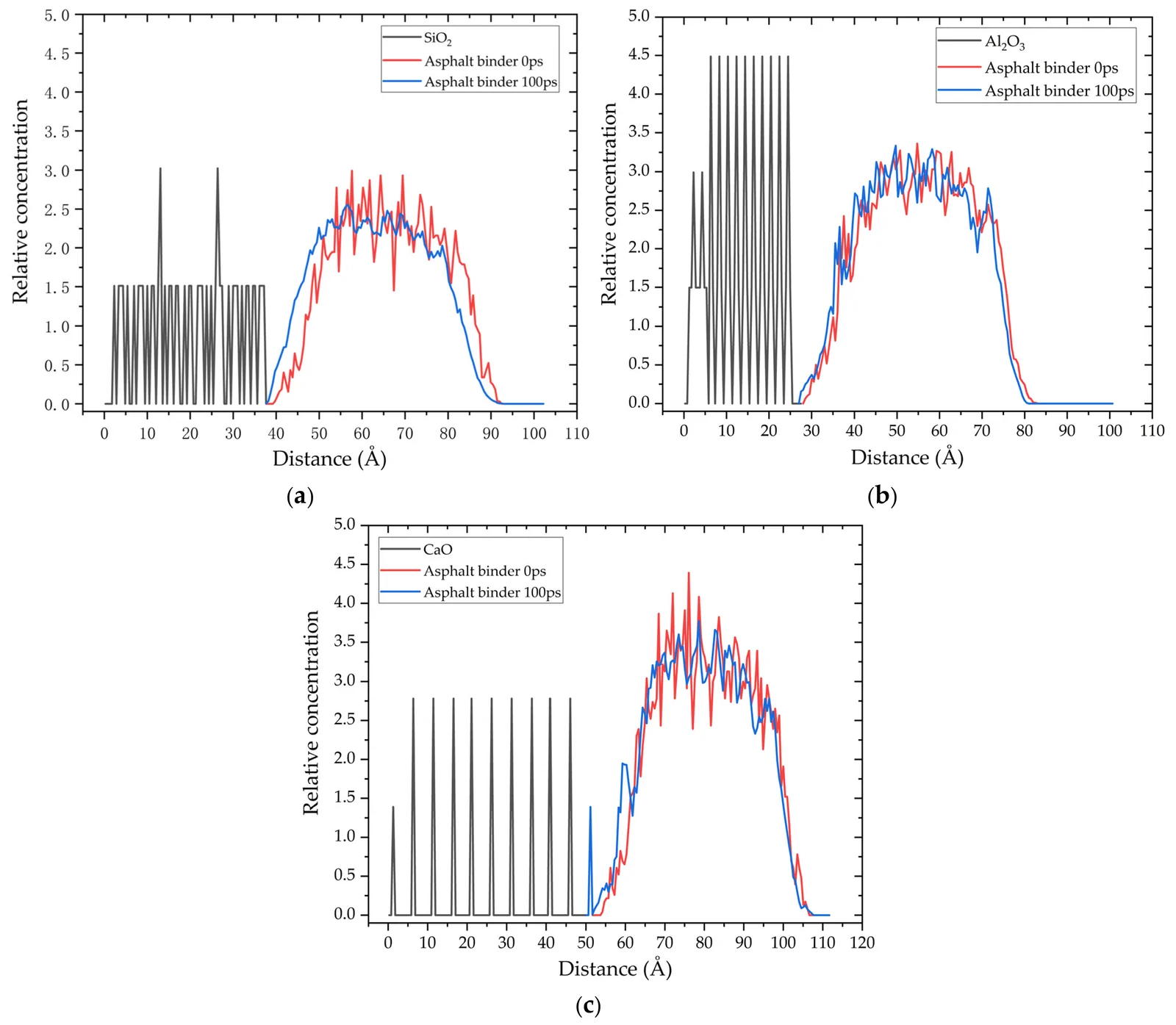
Relative concentration distribution profiles along z-axis under dry conditions: (**a**) asphalt–SiO_2_; (**b**) asphalt–Al_2_O_3_; (**c**) asphalt–CaO.

To investigate the migration behavior of asphalt molecules along the normal direction of the aggregate surface, the relative concentration distribution profiles along the z-axis were plotted, as shown in Figure 11. Since the aggregate atoms were constrained during the simulation, their distribution regions remained constant (0–38 Å for SiO_2_, 0–26 Å for Al_2_O_3_, and 0–50 Å for CaO). For the asphalt–SiO_2_ interface, asphalt molecules were predominantly distributed within the approximate range of 40–92 Å at the initial stage of the simulation. After 100 ps, the entire distribution region of asphalt molecules shifted notably toward the SiO_2_ aggregate surface, indicating that asphalt molecules gradually migrated and accumulated toward the aggregate during the simulation. Similar migration behavior was also observed for asphalt molecules on both Al_2_O_3_ and CaO surfaces. These results demonstrated that effective interfacial interactions existed between the asphalt binder and aggregate, driving the spontaneous enrichment of asphalt molecules in the interfacial region.

**Figure 12:**
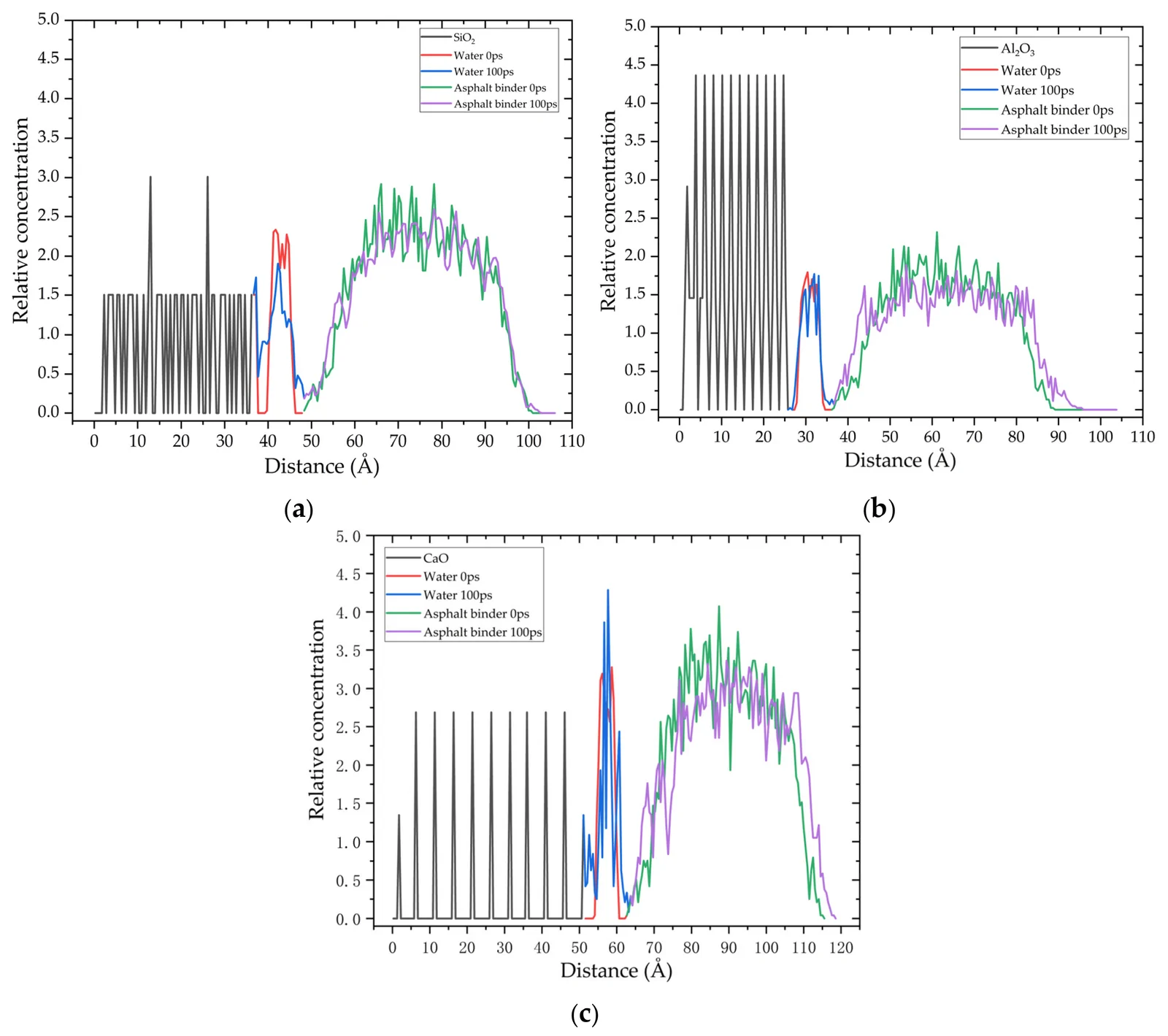
Relative concentration distribution profiles along z-axis under wet conditions: (**a**) asphalt–water–SiO_2_; (**b**) asphalt–water–Al_2_O_3_; (**c**) asphalt–water–CaO.

The relative concentration distribution profiles at the interface between asphalt binder and wet aggregate are shown in Figure 12. In the initial state, the concentration profiles can be clearly divided into three distinct regions along the z-axis, corresponding to the characteristic distribution zones of the aggregate, water, and asphalt binder, respectively. After 100 ps, the concentration profiles underwent significant changes. The sharp phase boundaries at the original interface regions no longer existed. Instead, an interfacial transition layer with continuous compositional gradients was formed. The concentration curves at the interface became smoother, and the width of the transition zone increased notably. Specifically, water molecules moved toward the aggregate surface, while some water molecules entered the asphalt interfacial region, causing slight swelling of the asphalt binder. It is worth noting that the thickness of the water film on the CaO surface was considerably greater than that on SiO_2_ and Al_2_O_3_. This is due to the stronger hydrophilicity and hydration tendency of the alkaline CaO surface, leading to a thicker hydration layer that more strongly hinders asphalt–aggregate contact and aggravates moisture-induced adhesion loss.

### 3.3. Interfacial Adhesion Work

#### 3.3.1. Temperature Effect

**Figure 13:**
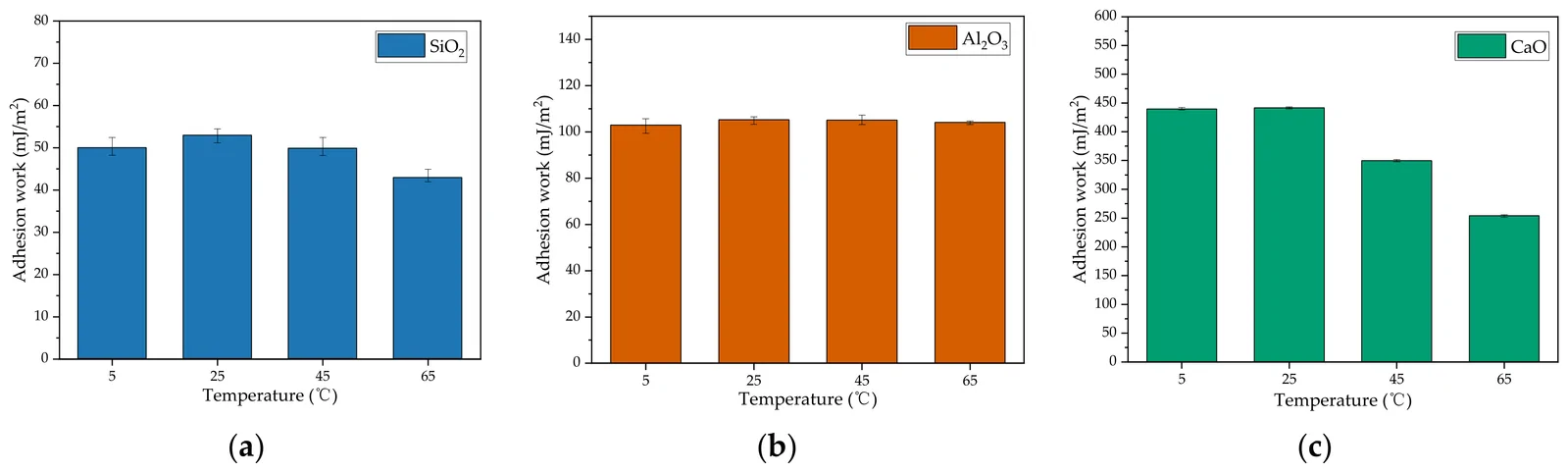
Variation in adhesion work with temperature under dry conditions: (**a**) SiO_2_; (**b**) Al_2_O_3_; (**c**) CaO.

The adhesion work for the asphalt–aggregate interfaces at different temperatures is presented in Figure 13. The adhesion work of the three asphalt–aggregate interfaces decreased in the order of CaO, Al_2_O_3_, and SiO_2_. Notably, the adhesion work between the asphalt binder and CaO was significantly higher than that for the other two aggregates due to the acid–base interactions between the lattice oxygen on the alkaline oxide surface and the polar functional groups in the asphalt binder. In contrast, the SiO_2_ surface, dominated by covalent bonds and weak polarity, relied mainly on physical adsorption, thus yielding the lowest adhesion work.

With rising temperature, the adhesion work first increased and then decreased, indicating a dual effect of temperature on interfacial bonding. In the low-temperature range, thermal energy enhanced the mobility of asphalt molecules and their wetting on the aggregate surface, thereby increasing the effective contact area and enhancing interfacial bonding. However, when the temperature exceeded 25 °C, excessive thermal motion degraded adhesion by weakening non-bonding interactions and promoting molecular desorption, which together reduced the effective contact area at the interface. The temperature-dependent trend of adhesion work is also supported by previous experimental findings, confirming the reliability of the simulation results [28,29,30].

#### 3.3.2. Moisture Effect

**Figure 14:**
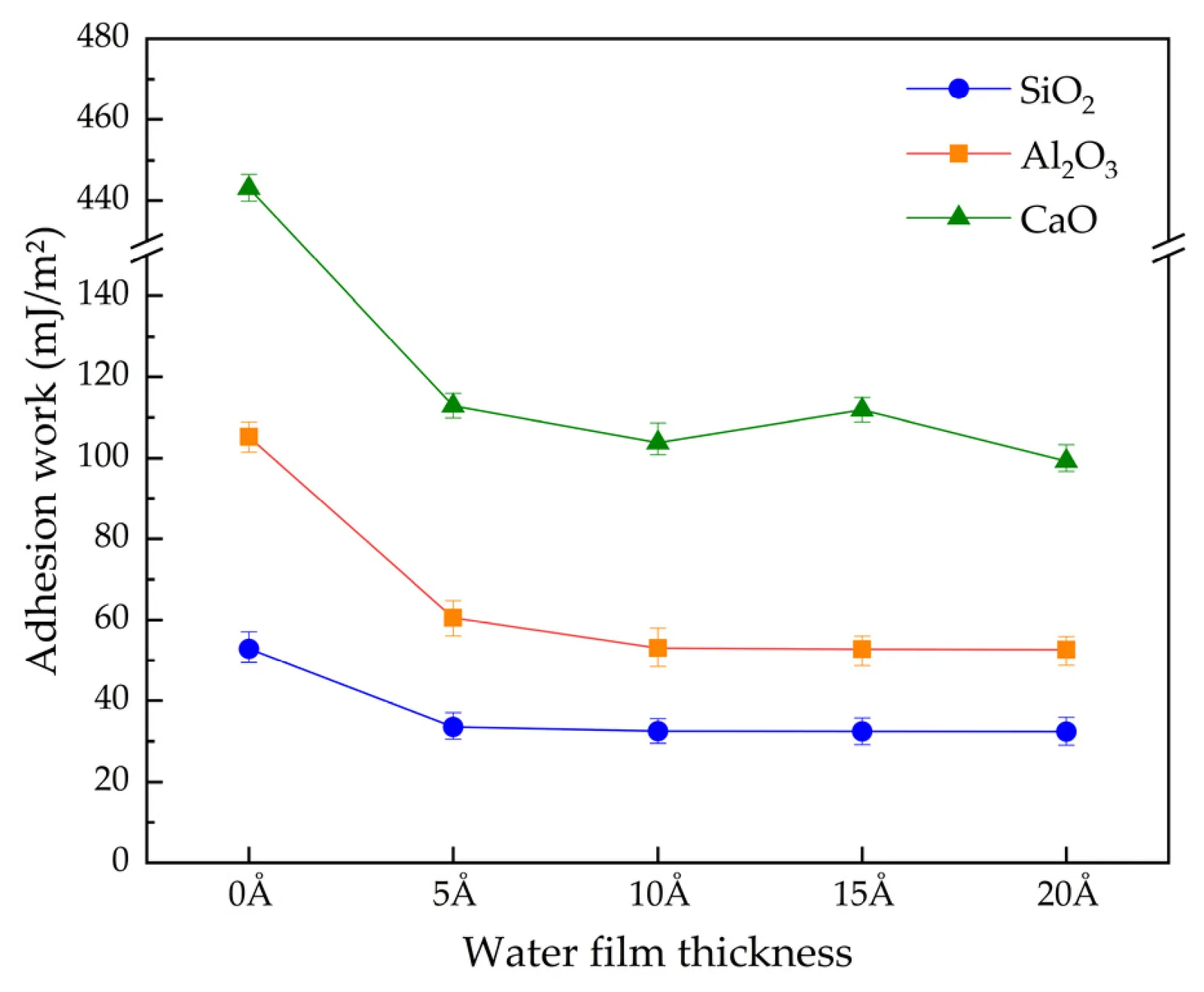
Effect of moisture on the adhesion work.

Figure 14 shows the adhesion work of the asphalt–aggregate interfaces under dry and wet conditions at 25 °C. Compared with the dry state (0 Å), the presence of water (5 Å) reduced the adhesion work obviously, with CaO, Al_2_O_3_, and SiO_2_ decreasing by approximately 75%, 51%, and 39%, respectively. Among them, CaO showed the largest decrease, consistent with the observation of a thicker water film observed on its surface. These results indicate that although CaO exhibited the best adhesion performance under dry conditions, it was also the most moisture-sensitive, thus posing a higher risk of moisture damage. However, the adhesion work showed only minor variations with increasing water film thickness from 5 Å to 20 Å. This might be attributed to the treatment of water and aggregate as an integrated composite phase, where the asphalt binder contacted the water-covered aggregate surface. Increasing the water film thickness did not substantially alter the composite surface. This trend is consistent with the moisture damage behavior reported in previous studies [31].

#### 3.3.3. Coupled Effects of Moisture and Temperature

**Figure 15:**
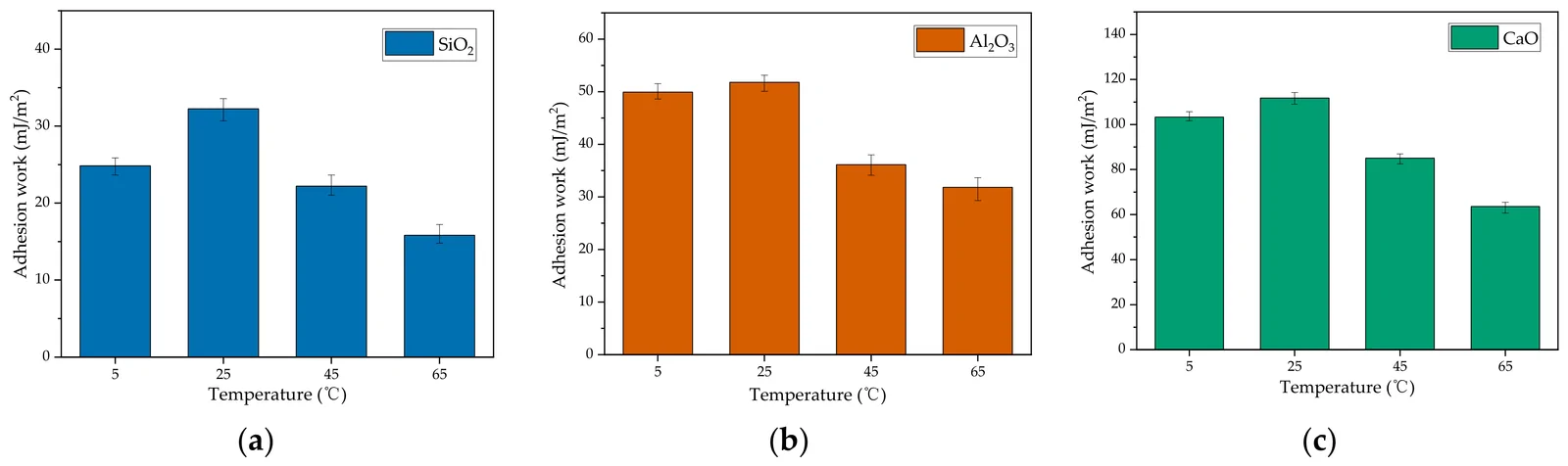
Variation in adhesion work with temperature under wet conditions: (**a**) SiO_2_; (**b**) Al_2_O_3_; and (**c**) CaO.

The adhesion work under the coupled effects of moisture and temperature is presented in Figure 15. Compared with the dry-state adhesion work in Figure 13, the wet-state adhesion work decreased substantially, yet it still exhibited a temperature-dependent trend of first increasing and then decreasing. Notably, under dry conditions, the adhesion work between asphalt binder and CaO showed the smallest variation with temperature (approximately 18%), whereas under wet conditions it exhibited a larger fluctuation (a decrease of about 33% from 25 °C to 65 °C). This indicates that the combined effects of moisture and temperature led to a greater reduction in adhesion work. This observation is consistent with the experimental findings of Zhang et al. [32], who reported deterioration in asphalt–aggregate interfacial performance under the combined effects of moisture and high temperature through peeling and Wilhelmy plate tests.

**Figure 16:**
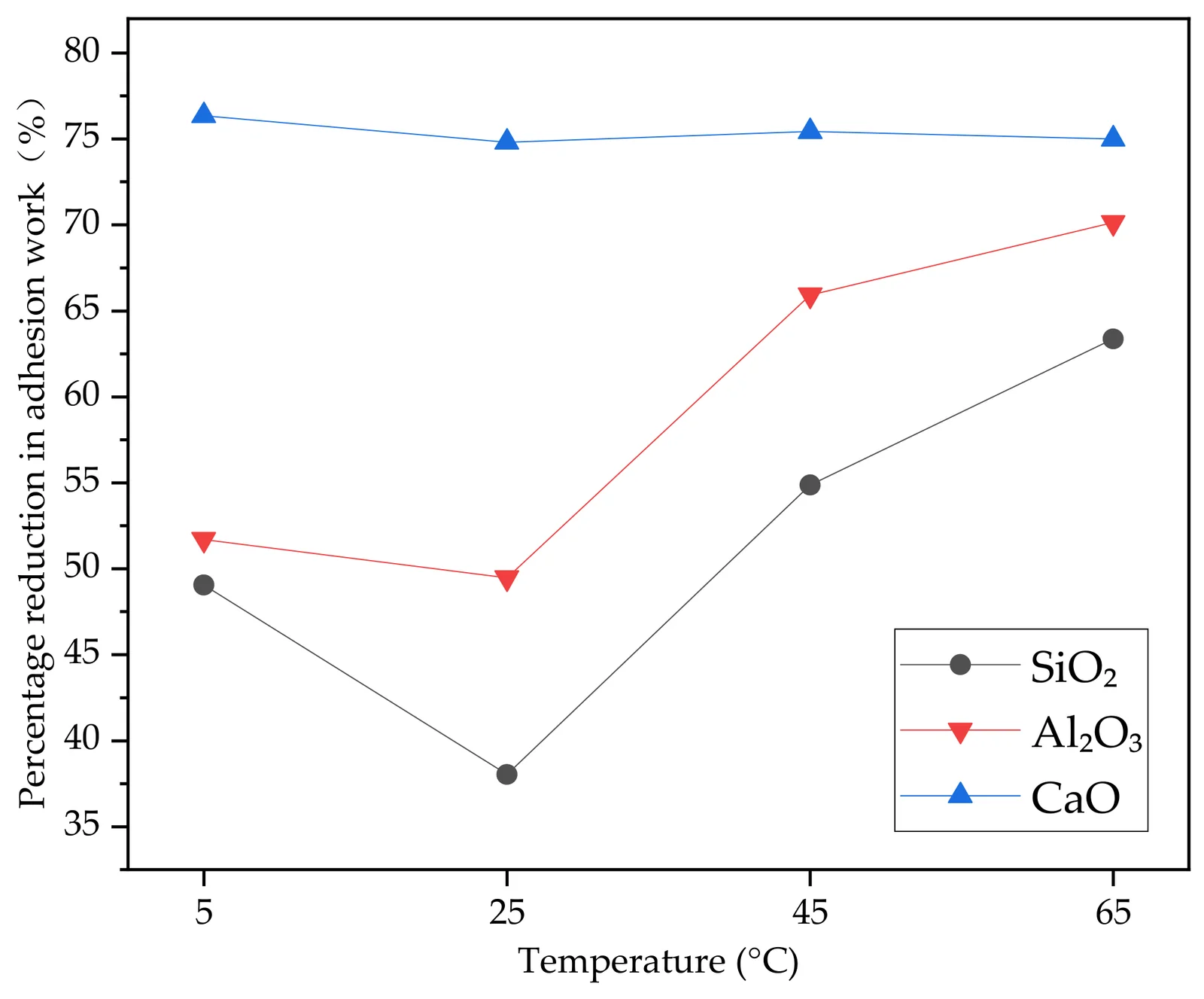
Variation in percentage reduction in adhesion work.

The percentage reduction in adhesion work is defined as the decrease in adhesion work from the dry-state interface to the wet-state interface (5 Å water film), which is expressed as a percentage of the dry-state adhesion work. The calculation results are depicted in Figure 16. After introducing a 5 Å water film, the adhesion work decreased substantially for all interfaces. In terms of the percentage reduction, CaO exhibited the highest reductions with relatively weak temperature dependence, indicating that the CaO interface is highly moisture-sensitive, but its relative loss is less affected by temperature. In contrast, SiO_2_ and Al_2_O_3_ showed more pronounced temperature-dependent variations. Their percentage reductions reached minima at 25 °C and increased markedly at higher temperatures, especially at 65 °C (63.43% for SiO_2_ and 70.02% for Al_2_O_3_). This indicated that the coupled high-temperature and -moisture effects more severely deteriorated the adhesion performance of SiO_2_ and Al_2_O_3_ interfaces, whereas the CaO interface suffered a consistently high-moisture-induced loss regardless of temperature.

### 3.4. Comparative Assessment of Factor Influences

A three-way analysis of variance (ANOVA) was performed to quantify the relative contributions of aggregate composition, temperature, and moisture to the adhesion work. The factors were aggregate composition (SiO_2_, Al_2_O_3_, and CaO), temperature (5 °C, 25 °C, 45 °C, and 65 °C), and moisture (dry and wet). The dependent variable was the adhesion work (mJ/m^2^). The effect size was reported as η^2^, calculated as the ratio of the sum of squares for each source to the total sum of squares. The ANOVA results are listed in Table 2.

The ANOVA results show that aggregate composition was the most influential factor, accounting for 47.2% of the total variance in adhesion work (η^2^ = 0.472, *p* < 0.001). Moisture was the second most important factor, explaining 24.8% of the variance (η^2^ = 0.248, *p* < 0.001). The moisture × aggregate composition interaction was also significant and substantial (η^2^ = 0.210, *p* < 0.001), indicating that the moisture sensitivity of the interface depends strongly on the aggregate composition. In contrast, temperature alone had a relatively small effect, accounting for only 2.4% of the variance (η^2^ = 0.024, *p* = 0.111). The interactions of temperature with moisture and aggregate composition were not statistically significant, with small combined contributions. These results suggested that adhesion work was governed primarily by aggregate compositions, with moisture as the principal external reducing factor. Temperature had only a limited influence on adhesion work within the investigated range.

## 4. Conclusions

In this study, molecular models of asphalt–aggregate interface were constructed to investigate the interaction between asphalt binder and aggregate under coupled moisture and temperature effects. The main conclusions drawn are as follows:

(1) Asphalt molecules underwent adsorption and migration toward the aggregate surface, indicating a pronounced adsorption effect of the aggregate surface on asphalt components. Under both dry and baseline wet conditions, the calculated adhesion work of the three asphalt–aggregate interfaces followed the descending order of CaO > Al_2_O_3_ > SiO_2_.
(2) Aggregate surface chemistry dominated asphalt–aggregate interfacial adhesion work. Within the temperature range of 5 °C to 65 °C, the adhesion work of asphalt–aggregate interfaces first increased and then decreased, reaching a peak at 25 °C.
(3) The presence of water significantly lowered asphalt–aggregate interfacial adhesion. CaO exhibited the most pronounced reduction in adhesion and the highest moisture sensitivity, whereas the adhesion work varied only slightly with increasing water film thickness from 5 Å to 20 Å.
(4) The coupled effects of moisture and temperature degraded the adhesion at the asphalt–aggregate interface. CaO showed persistently high moisture sensitivity, with its relative adhesion loss remaining insensitive to temperature. SiO_2_ and Al_2_O_3_ experienced more pronounced adhesion loss under the combined action of elevated temperature and moisture.
(5) Aggregate composition and moisture primarily controlled interfacial adhesion work, with aggregate properties dictating the interface’s moisture sensitivity, whereas temperature showed limited influence under the tested conditions.

## Figures and Tables

**Table 1:** Details of asphalt molecular models.

Fraction	Code	Molecular Formula	Number	Mass Percentage (%)
Asphaltenes	a	C_66_H_81_N	6	7.41
	b	C_42_H_54_O	6	4.79
	c	C_51_H_62_S	4	3.93
Resins	d	C_40_H_59_N	8	6.16
	e	C_36_H_57_N	10	7.00
	f	C_18_H_10_S_2_	8	3.23
	g	C_40_H_60_S	30	23.89
	h	C_29_H_50_O	8	4.61
Aromatics	i	C_35_H_44_	22	14.21
	j	C_30_H_46_	26	14.70
Saturates	k	C_35_H_62_	8	5.37
	l	C_30_H_62_	8	4.70

**Table 2:** Three-way ANOVA results for the adhesion work.

Source	Sum of Squares	df	Mean Square	F	*p*	η^2^
Moisture	89,924.0	1	89,924.0	96.44	<0.001	0.248
Aggregate composition	171,243.9	2	85,621.9	91.83	<0.001	0.472
Temperature	8537.9	3	2846.0	3.05	0.111	0.024
Moisture × aggregate composition	76,138.9	2	38,069.4	40.83	<0.001	0.210
Moisture × temperature	1562.6	3	520.9	0.56	0.660	0.004
Aggregate composition × temperature	10,001.0	6	1666.8	1.79	0.241	0.028
Error (three-way interaction)	5594.6	6	932.4			0.015
Total	363,002.9	23				1.000

Notes: *p* < 0.05 indicates statistical significance. η^2^ represents the proportion of total variance explained by each source. Due to rounding, the sum of η^2^ may not equal exactly 1.000.

## Data Availability

The original contributions presented in this study are included in the article. Further inquiries can be directed to the corresponding author.
